# 2076. Intestinal colonization with multidrug-resistant organisms, horizontal transmission and risk for bloodstream infection

**DOI:** 10.1093/ofid/ofad500.146

**Published:** 2023-11-27

**Authors:** Polyxeni Karakosta, Georgios Meletis, Elisavet Kousouli, Efthymia Protonotariou, Sophia Vourli, Panagiota Christina Georgiou, Vasiliki Mamali, Lemonia Skoura, Olympia Zarkotou, Spyros Pournaras

**Affiliations:** Attikon University General Hospital, Medical School, National and Kapodistrian University of Athens, Athens, Greece, Athens, Attiki, Greece; AHEPA University Hospital, Medical School, Faculty of Health Sciences, Aristotle University of Thessaloniki, Thessaloniki, Greece., Thessaloniki, Thessaloniki, Greece; Tzaneio General Hospital of Piraeus, Athens, Greece., Piraeus, Attiki, Greece; AHEPA University Hospital, Medical School, Faculty of Health Sciences, Aristotle University of Thessaloniki, Thessaloniki, Greece., Thessaloniki, Thessaloniki, Greece; National and Kapodistrian University of Athens, Athens, Zakinthos, Greece; Attikon University General Hospital, Medical School, National and Kapodistrian University of Athens, Athens, Greece, Athens, Attiki, Greece; Tzaneio General Hospital of Piraeus, Piraeus, Attiki, Greece; AHEPA University Hospital, Medical School, Faculty of Health Sciences, Aristotle University of Thessaloniki, Thessaloniki, Greece., Thessaloniki, Thessaloniki, Greece; Tzaneio General Hospital of Piraeus, Piraeus, Attiki, Greece; Attikon University General Hospital, Medical School, National and Kapodistrian University of Athens, Athens, Greece, Athens, Attiki, Greece

## Abstract

**Background:**

The association between rectal colonization and subsequent bloodstream infections (BSI) by multidrug-resistant organisms (MDROs) remains to be clarified. We aimed to investigate the relative risk of BSI in rectal carriers of MDROs and in the case of carbapenem-resistant *Enterobacteriaceae (CRE),* whether BSI was due to the patient’s own colonizing strain.

**Methods:**

We retrospectively included all adult inpatients hospitalized from Jan 2019 to Dec 2022, in ICUs or medical wards of 3 Greek hospitals (Athens: “Attikon” Hospital, Piraeus: Tzaneio Hospital, Thessaloniki: AHEPA Hospital), who were screened for intestinal MDRO carriage at admission and weekly. Screening was performed by selective media and immunochromatography for the carbapenemase in CRE. Patients were followed up for BSI by the same colonizing organism; relative risk (95% CI) was estimated after adjustment for ICU hospitalization and age. MDROs included CRE, carbapenem-resistant *Acinetobacter baumannii* (CRAB), carbapenem-resistant *Pseudomonas aeruginosa* (CRPA) and vancomycin-resistant *enterococci* (VRE). The CRE-colonizers and *BSI*-pathogens were analyzed for carbapenemase type to identify potential similarities.

**Results:**

Of 4,427 patients screened, 1,321 (29.8%) were colonized by CRE, 1,255 (28.3%) with CRAB, 252 (5.7%) with CRPA and 859 (27.9% of 3,082 patients tested) with VRE. Of those, 207 (15.7%), 247 (19.7%), 23 (9.1%) and 30 (3.5%) subsequently developed CRE-BSI, CRAB-BSI, CRPA-BSI and VRE-BSI, respectively. Intestinal CRE colonization was associated with 4-fold higher risk of subsequent CRE-BSI, after adjusted for ICU stay and age [RR (95%CI): 4.1 (3.2, 5.2)]. The respective relative risk (95%CI) for BSI in patients with colonization by CRAB, CRPA and VRE were 2.3 (1.9, 2.8), 8.2 (5.1, 13.4) and 2.1 (1.3, 3.4). To evaluate possible horizontal transmission, 286 paired CRE strains from 143 patients were analyzed; in 111 (77.6%), both colonizers and *BSI* pathogens belonged to the same bacterial species and carried the same carbapenemase type.

**Table 1:**
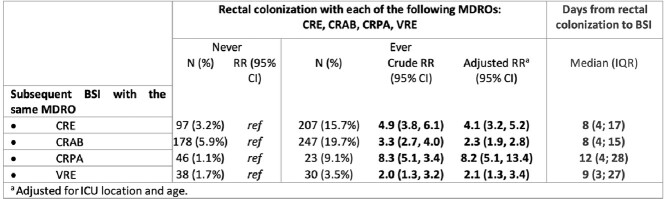
Rectal colonization with MDROs and the risk for BSI from the same pathogen

**Table 2:**
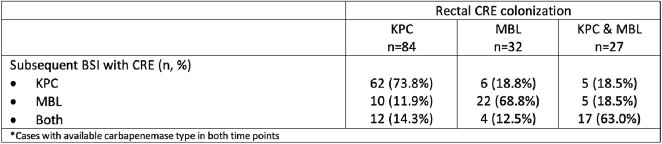
Carbapenemase types in rectal carriers of CRE who developed subsequent BSI with the same pathogen

**Conclusion:**

Intestinal colonization by MDROs was associated with increased risk of BSI by the colonizing bacteria. Clinicians should evaluate this information for empiric treatment, while genome-wide data are needed to substantiate horizontal transmission.

**Disclosures:**

**All Authors**: No reported disclosures

